# Supplementary material for: Deletion of wfs1 Impairs Oligodendrocyte Precursor Cells Dorsal Distribution and Myelination Through the wfs1-hmgcs1 Axis in Zebrafish
Source: Biology (Basel). 2026 Aug 16;15(16):1402. doi: 10.3390/biology15161402 (PMC13510151; doi:10.3390/biology15161402)
Supplement: Supplementary file 1 [file biology-15-01402-s001.zip › File S1-Transcriptome sequencing data and differentially expressed genes (DEGs) in wfs1-- zebrafish at 5 dpf/2.KEGG/wfs1KOvsWT/DOWN/src/dre04914.html]

dre04914


- dre:245702

- Down regulated genes

245702\_1(-1.45433245048201)

- dre:100000252

- Down regulated genes

100000252\_1(-1.7718817168434)

- dre:393223

- Down regulated genes

393223\_1(-2.70117970213935)

- dre:100000252

- Down regulated genes

100000252\_1(-1.7718817168434)

Close
